# Refractive lens power and lens thickness in children (6–16 years old)

**DOI:** 10.1038/s41598-021-98817-9

**Published:** 2021-09-29

**Authors:** Tailiang Lu, Jike Song, Qiuxin Wu, Wenjun Jiang, Qingmei Tian, Xiuyan Zhang, Jing Xu, Jianfeng Wu, Yuanyuan Hu, Wei Sun, Hongsheng Bi

**Affiliations:** 1 Shandong University of Traditional Chinese Medicine, No. 16369#, Jingshi Road, Jinan, 250014 People’s Republic of China; 2 Affiliated Eye Hospital of Shandong University of Traditional Chinese Medicine, No. 48#, Yingxiongshan Road, Jinan, 250002 People’s Republic of China; 3 Shandong Provincial Key Laboratory of Integrated Traditional Chinese and Western Medicine for Prevention and Therapy of Ocular Diseases, Key Laboratory of Integrated Traditional Chinese and Western Medicine for Prevention and Therapy of Ocular Diseases, Universities of Shandong, Eye Institute of Shandong University of Traditional Chinese Medicine, No. 48#, Yingxiongshan Road, Jinan, 250002 People’s Republic of China

**Keywords:** Epidemiology, Risk factors

## Abstract

To examine the refractive lens power (RLP) and lens thickness and their associated factors in children from North-Western China. Children from two schools (primary school and junior high school) in the North-Western Chinese province of Qinghai underwent a comprehensive ophthalmic examination including biometry and cycloplegic refractometry. The RLP was calculated using Bennett’s equation. The study included 596 (77.9%) individuals (mean age: 11.0 ± 2.8 years; range: 6–16 years) with a mean axial length of 23.65 ± 1.24 mm (range: 20.02–27.96 mm). Mean lens thickness was 3.30 ± 0.16 mm (range: 2.85–3.99 mm) and mean RLP was 24.85 ± 1.98D (range: 19.40–32.97). In univariate analysis, girls as compared to boys had a significantly thicker lens and greater RLP, shorter axial length, smaller corneal curvature radius and shorter corneal curvature radius (all *P* < 0.001). Both sexes did not differ significantly in refractive error (*P* = 0.11) and corneal thickness (*P* = 0.16). RLP was positively associated with refractive error (correlation coefficient r = 0.33; *P* < 0.001) and lens thickness (r = 0.62; *P* < 0.001) and negatively with axial length (r =  − 0.70; *P* < 0.001). In univariate analysis, RLP decreased significantly with older age in the age group from age 6–13, while it plateaued thereafter, with no significant difference between boys and girls. In multivariate regression analysis, a higher RLP was associated with younger age (*P* < 0.001; standard regression coefficient *β* =  − 0.07), female sex (*P* < 0.001; *β* =  − 0.08), shorter axial length (*P* < 0.001; *β* =  − 0.48) and higher lens thickness (*P* < 0.001; *β* = 0.42). In Chinese children, RLP with a mean of 24.85 ± 1.98D decreases with older age, male sex, longer axial length, and thinner lens thickness. Changes in RLP and axial length elongation are important players in the emmetropization and myopization.

## Introduction

The optical system of the eye undergoes marked changes in childhood and adolescence^1–10^. It includes changes in the corneal curvature, anterior chamber depth, anterior and posterior lens curvature and lens thickness, and vitreous chamber length. After the second year of life, the shape and dimensions of the cornea have mostly reached adult values, so that the lens, in particular the refractive power of the lens, in addition to the ocular axial length then plays a dominant role in the physiological process of emmetropization^3,11,12^.

The crystalline lens is a complex structure growing and changing grows throughout life^13,14^. Previous studies have shown that the refractive lens power (RLP) in children decreases in association with a decrease in lens thickness with older age in children^5,15–18^. Some longitudinal studies have suggested that the thinning of the lens decreased or stopped in the year when myopia started^9,10^. In adulthood, the RLP linearly decreases with age, with a steeper decline after the age of 50 years^10,19^.

The RLP cannot be measured directly due to the intraocular position of the lens. The RLP can be determined by phakometry, which measures the radius of the lens curvature by analyzing Purkinje image data^20,21^, or the RLP can be calculated from other ocular biometric measures. To cite an example, these calculations apply Bennett’s formula, assuming that the natural lens is like that lens of the Gullstrand-Emsley model eye^22–24^. Bennett used a thick-lens description that makes assumptions about the shape and refractive index distribution of the lens based on the Gullstrand-Emsley schematic eye^22,24^. On this basis, he calculated the equivalent of the RLP in a way validated by phakometry^23^.

Since the RLP in children and adolescents has not been fully explored in Chinese, since it was not assessed yet in children from West China, we conducted the present study to assess the distribution of the RLP and its associated factors in children from the Qinghai-Tibet Plateau.

## Methods

The cross-sectional school-based study enrolled a total of 765 children aged 6–16 years from two elementary schools (The Second Primary School of Menyuan County and Qingshizui Town Boarding School of Menyuan County) and one junior high school (The Third Junior High School of Menyuan County) in the Menyuan county in Qinghai province, China. The Ethics Committee of Shandong University of Traditional Chinese Medicine approved the study protocol, and all participants one parent or legal guardian signed a written informed consent. The study was conducted in accordance with the tenets of the Declaration of Helsinki.

The Menyuan Hui autonomous county in the Qinghai province is in the northeast of Qinghai province, with a total area of 6896 square kilometers at an altitude of 2388 to 5254 m above sea level. It has a continental plateau climate, with snow and wind in spring, cool and rainy summers, mild and short autumns, and cold and long winters. There are 22 ethnic groups in Menyuan county, including Hui, Han, Tibetan, Mongolian, and Tujia. Among these ethnicities, 62.4% belong to the so-called ethnic minorities and 45.8% of the people have the Hui nationality. Most of the population are farmers and herdsmen. The school-based study enrolled 596 children, of which 353 (59.2%) are Han Chinese, 183 (30.7%) were Hui, and 60 (10.1%) were Tibetan.

All participants underwent an ophthalmic examination including measurement of distance and near, uncorrected, and best corrected, visual acuity and cycloplegic refractometry. For cycloplegia, the children first received one drop of 0.4% oxybuprocaine (Santen Corp., Shiga, Japan) for topical anesthesia. Two minutes later, the first of three drops of 1% cyclopentolate (Alcon, Fort Worth, Tex., USA) were instilled at 5-min intervals. If 30 min later the pupil diameter was at least 6 mm, refractometry was performed. If the pupil diameter was < 6 mm, a fourth drop of cyclopentolate was applied. The spherical equivalent of refraction (SE) was defined as the spherical refractive power plus half of the cylindrical refractive power.

Using optical coherence interferometry (Lenstar LS900; Haag-Streit Co., Koeniz, Switzerland), we measured the axial length, corneal curvature radius, central corneal thickness, anterior chamber depth, lens thickness, and the vitreous chamber depth. All biometric measurements were performed five times, and the mean value of these measurements was taken for statistical analysis. The mean corneal power was calculated based on the mean corneal curvature radius with a refractive index of the cornea of 1.3315^25^. Applying the formula of Bennett, the RLP was calculated based on the cycloplegic refractive error, corneal power, anterior chamber depth, lens thickness, and axial length. The equation was as follows by the formula of Bennett^22,23^.

$$ {\text{P}}_{{{\text{L}},{\text{ Bennett}}}} = \frac{{ - 1000\,{\text{n(S}}_{{{\text{CV}}}} + K)}}{{1000{\text{n}} - {(}ACD + {\text{c}}_{{1}} T)(S_{CV} + K)}} + \frac{{1000{\text{n}}}}{{{\text{c}}_{{2}} T + V}} $$

In this formula, P_L, Bennett_ is the lens power using Bennett method, n = 4/3 the aqueous and vitreous index, S_CV_ is the spherical equivalent refraction at the corneal vertex, K is the mean corneal power, T is the lens thickness, and V is the vitreous depth. Effective anterior chamber depth (ACD) included central corneal thickness and ACD measured by LENSTAR. Bennett estimated the c_1_ = 0.596 and c_2_ =  − 0.358 constants using the Gullstrand-Emsley eye model.

With respect to the refractive status, we differentiated between high myopia (SE <  − 6.0D), myopia (SE ≤  − 0.5D), emmetropia (SE >  − 0.5D to ≤  + 0.5D), mild hyperopia (0.5D < SE ≤ 2.0D) and medium to marked hyperopia (SE > 2.0D)^26^. As the spherical equivalents of right and left eyes were highly correlated with each other, the data are presented for right eyes only.

The statistical analysis was conducted using a commercially available software (SPSS for Windows, version 25.0, IBM-SPSS, Chicago, Ill., USA). The mean values were presented as mean and standard deviations. Comparisons were performed using independent sample *t*-test between gender groups and with the analysis of variance (ANOVA) and post hoc tests for the refractive error groups. Correlations of the ocular biometric parameters were analyzed using the Pearson´s correlation coefficient. We assessed the standardized regression coefficient *β*, the non-standardized regression coefficient B and its 95% confidence intervals (CIs). The multivariate regression analysis included the parameters of RLP (or lens thickness) as the dependent variable and as independent variables all those parameters which were significantly associated with RLP (or lens thickness) in the univariate analyses. Statistical significance was considered with a *P*-value of less than 0.05.

## Results

Out of 765 eligible children, 596 (77.9%) individuals (288 [48.3%] girls) had a full set of data required for this study, so that the study eventually included 596 participants. The reason why 169 children eventually did not participate was that the parents refused cycloplegia for refractometry. The mean age of children was 11.0 ± 2.8 years old (range: 6 to 16 years old), mean refractive error was − 1.31 ± 2.41D (range − 11.63 to 8.75D), and the mean axial length was 23.65 ± 1.24 mm (range: 20.02 to 27.96 mm) (Table 1). The children with a full set of data as compared to those who did not undergo cycloplegic refractometry were significantly older (10.31 ± 2.41 versus 11.01 ± 2.85 years old; *P* = 0.004). They did not differ in sex (*P* = 0.89).

**Table 1:** General characteristics of the study participants stratified by sex.

Parameters	Boys (n = 308)	Girls (n = 288)	Total (n = 596)	t	*P*
Age (years)	11.07 ± 2.92	10.98 ± 2.75	11.03 ± 2.84	− 0.466	0.64
Axial length (mm)	23.89 ± 1.20	23.41 ± 1.24	23.65 ± 1.24	− 4.789	< 0.001
Spherical equivalent (D)	− 1.16 ± 2.18	− 1.47 ± 2.62	− 1.31 ± 2.41	− 1.585	0.11
Corneal curvature radius (mm)	7.83 ± 0.25	7.70 ± 0.26	7.77 ± 0.27	− 6.214	< 0.001
Corneal thickness (mm)	0.52 ± 0.03	0.51 ± 0.04	0.52 ± 0.03	− 1.412	0.16
Anterior camber depth (mm)	3.26 ± 0.25	3.16 ± 0.25	3.21 ± 0.25	− 4.813	< 0.001
Lens thickness (mm)	3.28 ± 0.17	3.32 ± 0.15	3.30 ± 0.16	2.843	0.005
Vitreous chamber depth (mm)	16.83 ± 1.15	16.41 ± 1.22	16.63 ± 1.20	− 4.273	< 0.001
Lens refractive power (D)	24.24 ± 1.79	25.51 ± 1.95	24.85 ± 1.98	8.311	< 0.001

**Figure 1:**
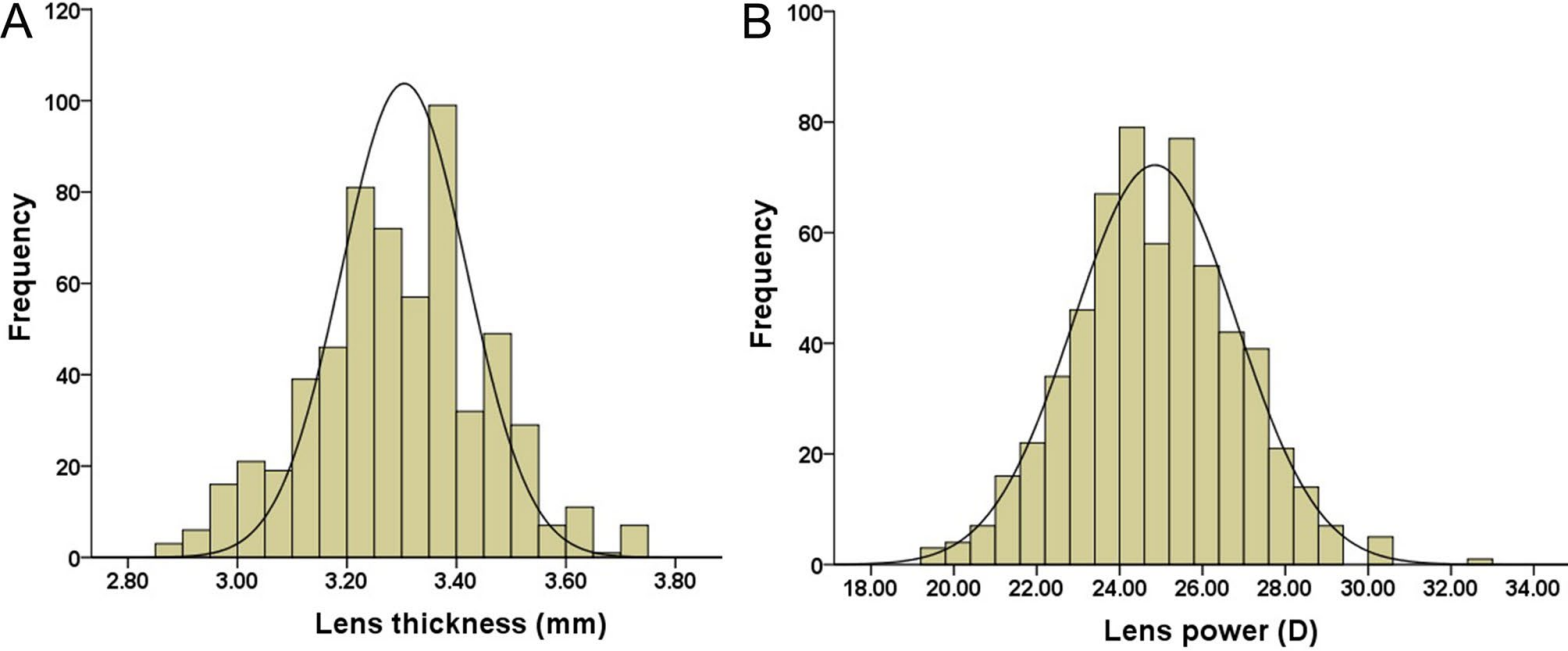
The distribution of lens thickness **(A)** and refractive lens power **(B)** in the right eyes of the study population.

The mean lens thickness was 3.30 ± 0.16 mm (range: 2.85–3.99 mm) (Fig. 1A), and the mean RLP was 24.85 ± 1.98D (range: 19.40 to 32.97D) (Fig. 1B). Lens thickness and RLP were normally distributed (*P* < 0.001, Kolmogorov–Smirnov test).

**Figure 2:**
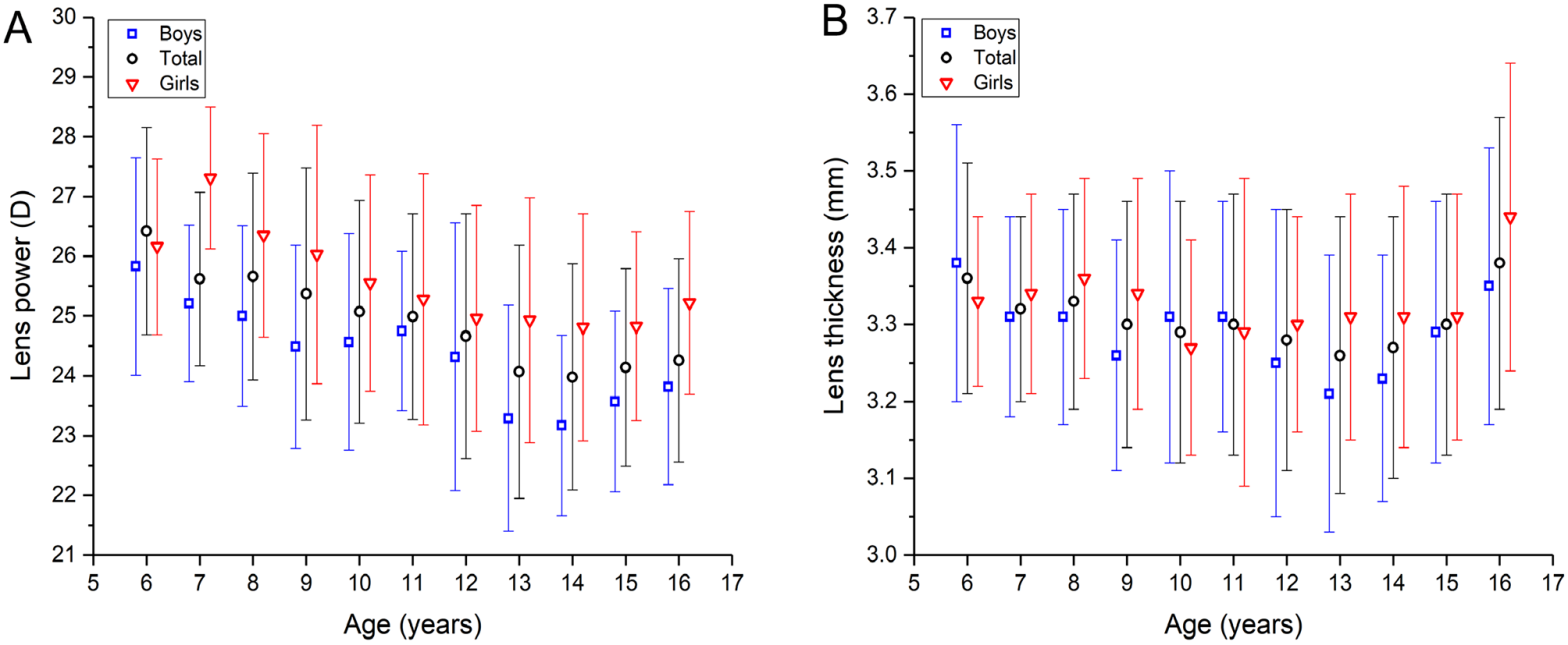
Distribution of lens thickness **(A)** and refractive lens power **(B)** in the Qinghai Children Eye Study, stratified by age and gender.

**Figure 3:**
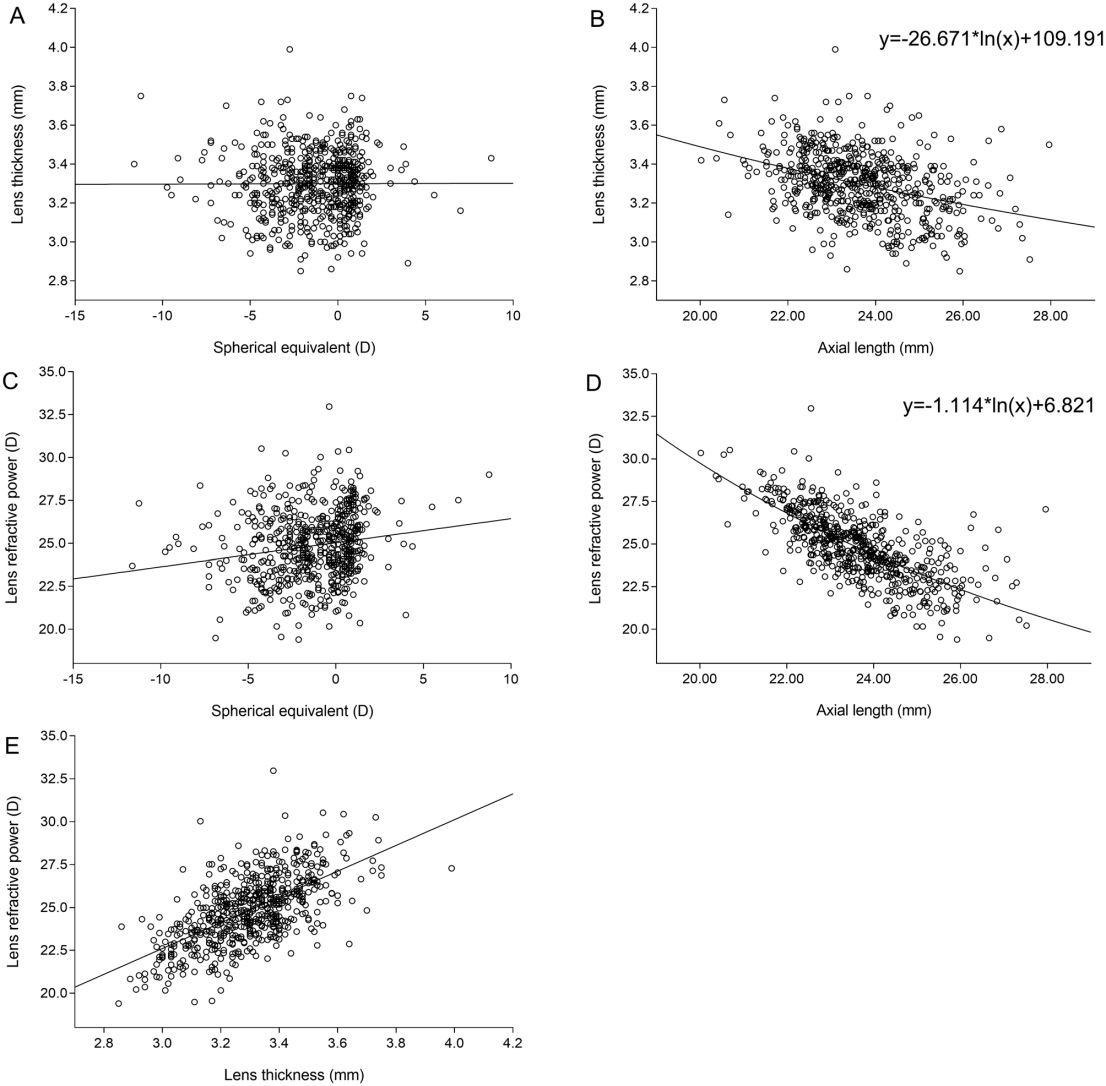
Graph showing the association between lens thickness and refractive error **(A)**; the logarithmic curve-fitting showing the association between lens thickness and axial length **(B)**; graph showing the association between refractive lens power and refractive error **(C)**; the logarithmic curve-fitting showing the association between refractive lens power and axial length **(D)**; graph showing the association between refractive lens power and lens thickness **(E)** .

In univariate analysis, girls as compared to boys had a significantly thicker lens and greater RLP, shorter axial length, smaller corneal curvature radius and shorter (all *P* < 0.001). Both sexes did not differ significantly in refractive error (*P* = 0.11) and corneal thickness (*P* = 0.16) (Table 1). Lens thickness decreased from age 6 to 13 years old and increased after the age of 13 years old in boys and 15 years old in girls (Fig. 2A). We used independent sample *t*-test compare the lens thickness between girls and boys. The data exhibited that lens thickness was significantly greater in girls than in boys in the age of 9 and 13 years old, while in the other age groups, both sexes did not differ in lens thickness (Table 2). There were no significant differences in lens thickness between the various ethnic groups (*P* = 0.68). Lens thickness was highest (*P* < 0.001) in children with medium to marked hyperopia, followed by children with mild hyperopia and emmetropic children, and finally myopic children. Lens thickness was positively correlated with refractive error (r = 0.20; *P* < 0.001) and negatively with axial length (r =  − 0.36; *P* < 0.001) (Fig. 3).

**Table 2:** The Lens thickness (mean ± standard deviation) and refractive lens power (mean ± standard deviation) in the Qinghai Children Eye Study, stratified by age, gender and ethnicity.

Age (years)	n	Lens thickness (mm)				Refractive lens power (Diopter)			
		Boys	Girls	Total	*P*	Boys	Girls	Total	*P*
6	20	3.38 ± 0.18	3.34 ± 0.13	3.36 ± 0.15	0.65	25.83 ± 1.82	27.31 ± 1.19	26.42 ± 1.73	0.059
7	51	3.31 ± 0.13	3.33 ± 0.11	3.32 ± 0.12	0.46	25.21 ± 1.31	26.16 ± 1.47	25.62 ± 1.45	0.02
8	70	3.31 ± 0.14	3.36 ± 0.13	3.33 ± 0.14	0.12	25.00 ± 1.51	26.35 ± 1.70	25.66 ± 1.73	0.001
9	79	3.26 ± 0.15	3.34 ± 0.15	3.30 ± 0.16	0.02*	24.49 ± 1.70	26.03 ± 2.16	25.37 ± 2.11	0.001
10	65	3.31 ± 0.19	3.27 ± 0.14	3.29 ± 0.17	0.35	24.57 ± 1.81	25.55 ± 1.81	25.07 ± 1.86	0.03
11	46	3.31 ± 0.15	3.29 ± 0.20	3.30 ± 0.17	0.74	24.75 ± 1.33	25.28 ± 21.0	24.99 ± 1.72	0.31
12	30	3.25 ± 0.20	3.30 ± 0.14	3.28 ± 0.17	0.42	24.32 ± 2.24	24.96 ± 1.89	24.66 ± 2.05	0.40
13	76	3.21 ± 0.18	3.31 ± 0.16	3.26 ± 0.18	0.02*	23.29 ± 1.89	24.93 ± 2.05	24.07 ± 2.12	0.001
14	81	3.23 ± 0.16	3.31 ± 0.17	3.27 ± 0.17	0.05	23.17 ± 1.51	24.81 ± 1.90	23.98 ± 1.89	< 0.001
15	59	3.29 ± 0.17	3.31 ± 0.16	3.30 ± 0.17	0.76	23.57 ± 1.51	24.83 ± 1.58	24.14 ± 1.65	0.003
16	19	3.35 ± 0.18	3.44 ± 0.20	3.38 ± 0.19	0.36	23.82 ± 1.64	25.22 ± 1.53	24.26 ± 1.70	0.09
Total	596	3.28 ± 0.17	3.32 ± 0.15	3.30 ± 0.16	0.005*	24.24 ± 1.79	25.51 ± 1.95	24.85 ± 1.98	< 0.001
**Ethnicity**									
Han	353	3.28 ± 0.17	3.31 ± 0.16	3.29 ± 0.16		24.26 ± 1.81	25.53 ± 1.96	24.88 ± 1.99	
Hui	183	3.28 ± 0.17	3.34 ± 0.14	3.31 ± 0.16		24.21 ± 1.85	25.51 ± 1.99	24.83 ± 2.02	
Tibetan	60	3.29 ± 0.16	3.31 ± 0.17	3.30 ± 0.17		24.16 ± 1.62	25.37 ± 1.80	24.74 ± 1.80

In a similar manner, RLP was positively associated with refractive error (r = 0.33; *P* < 0.001) and negatively with axial length (r =  − 0.70; *P* < 0.001). RLP was positively correlated with lens thickness (r = 0.62; *P* < 0.001) (Fig. 3).

To exclude the effect of corneal refraction, partial correlation of the SPSS was used to study the relationship between axial length and lens thickness or RLP. RLP and lens thickness was associated with axial length (r =  − 0.693, *P* < 0.001; r =  − 0.347, *P* < 0.001).

The logarithmic curve revealed that the lens thickness decreased significantly with elongation of axial length, as demonstrated by the equation from the logarithmic curve-fitting regression analysis (*P* < 0.001), Y =  − 26.671*ln(x) + 109.191, with a coefficient of determination R^2^ of 0.129 [axial length, x (mm); lens thickness, y (mm)] (Fig. 3B). In a similar manner, the RLP decreased significantly with elongation of axial length, as demonstrated by the equation from the logarithmic curve-fitting regression analysis (P < 0.001), Y =  − 1.114*ln(x) + 6.821, with a coefficient of determination R^2^ of 0.498 [axial length, x (mm); RLP, y (D)] (Fig. 3D).

In the multivariate regression analysis with the parameter of lens thickness as the dependent variable, we first dropped refractive error due to a collinearity with axial length (variance inflation factor [VIP]: 9.87). We then removed step by step from the list of independent variables those parameters for which the *P*-value was > 0.05. In the final model, a higher lens thickness was associated with older age (*P* < 0.001, *β* = 0.009), shorter axial length (*P* < 0.001; *β* =  − 0.052), longer corneal curvature radius (*P* < 0.001; *β* = 0.115), and shorter anterior chamber depth (*P* < 0.001; *β* =  − 0.145) (Table 3).

**Table 3:** Multivariate linear regression analysis of associations between lens thickness and ocular and systemic parameters in the Qinghai Children Eye Study.

Parameters	*P*	Standardized regression coefficient beta	Unstandardized regression coefficient B	95% CI of B	Variance inflation factor
Age	< 0.001	0.009	0.161	0.004 ,0.014	1.467
Axial length	< 0.001	− 0.052	− 0.399	− 0.066, − 0.038	2.252
Corneal curvature radius (mm)	< 0.001	0.115	0.188	0.064, 0.167	1.392
Anterior chamber depth (mm)	0.013	− 0.145	− 0.226	− 0.199, − 0.091	1.384

In univariate analysis, RLP decreased significantly with older age in the age group from age 6 to age 13 (correlation coefficient r =  − 0.34; *P* < 0.001), while it plateaued in the age group of 13 to 16 years (Table 2) (Fig. 2B), with no significant difference between boys and girls. There were no significant differences in RLP between the ethnic groups (*P* = 0.88). RLP, without significant difference between boys and girls, was significantly (*P* < 0.001) higher than in myopic children (Table 4). It did not differ significantly between myopic children and highly myopic children or between children with medium to marked hyperopia and mild hyperopia (*P*˃0.05).

**Table 4:** Lens thickness and refractive lens power stratified by the refractive status and sex.

Refractive status	Lens thickness (mm)				Refractive lens power (Diopter)			
	Boys	Girls	Total	*P*	Boys	Girls	Total	*P*
Medium to marked hyperopia (spherical equivalent [SE] > 2.0D diopters)	3.39 ± 0.18	3.46 ± 0.15	3.43 ± 0.16	0.49	25.54 ± 1.19	28.13 ± 2.33	27.05 ± 2.30	0.047
Mild hyperopia (0.5 < spherical equivalent [SE] ≤ 2.0D)	3.33 ± 0.15	3.35 ± 0.15	3.34 ± 0.15	0.51	25.22 ± 1.68	26.36 ± 1.52	25.70 ± 1.71	< 0.001
Emmetropia (− 0.5D < spherical equivalent [SE] ≤ + 0.5D)	3.31 ± 0.15	3.34 ± 0.14	3.32 ± 0.15	0.18	24.70 ± 1.35	26.16 ± 1.65	25.33 ± 1.65	< 0.001
Moderate myopia (− 6.0D ≤ spherical equivalent [SE] ≤ − 0.5D)	3.25 ± 0.17	3.29 ± 0.16	3.27 ± 0.16	0.02	23.55 ± 1.74	24.89 ± 1.93	24.24 ± 1.96	< 0.001
High myopia (spherical equivalent [SE] < − 6.0D)	3.13 ± 0.15	3.34 ± 0.14	3.27 ± 0.17	0.004	23.10 ± 1.65	25.37 ± 1.55	24.54 ± 1.91	< 0.001
			F = 7.897, P < 0.001				F = 21.97, P < 0.001	

In the multivariate regression analysis with the parameter of RLP as the dependent variable, we first dropped refractive error due to a collinearity with axial length (variance inflation factor (VIP): 11.87). We then removed step by step from the list of independent variables those parameters for which the *P*-value was > 0.05. In the final model, a higher RLP was associated with younger age (*P* < 0.001, *β* =  − 0.07), female sex (*P* < 0.001; *β* =  − 0.08), shorter axial length (*P* < 0.001; *β* =  − 0.483) and higher lens thickness (*P* < 0.001; *β* = 0.42) (Table 5).

**Table 5:** Multivariate linear regression analysis of associations between refractive lens power and ocular and systemic parameters in the Qinghai Children Eye Study.

Parameters	*P*	Standardized regression coefficient beta	Unstandardized regression coefficient B	95% CI of B	Variance inflation factor
Axial length (mm)	< 0.001	− 0.483	− 0.77	− 0.86, − 0.68	1.54
Lens thickness (mm)	< 0.001	0.421	5.12	4.52, 5.72	1.16
Age (years)	0.01	− 0.067	− 0.05	− 0.08, − 0.01	1.32
Gender	< 0.001	− 0.08	− 0.71	− 0.90, − 0.53	0.05

## Discussion

In our study population of Chinese children from the West Chinese Qinghai Province, a higher RLP was associated with younger age, female sex, shorter axial length, and larger lens thickness. The mean RLP was 24.9 ± 2.0D.

The results of our study agree with observations made in previous investigations, including the age-related decline in lens thickness and RLP^1–5,7,8,14,18,27–29^. Longitudinal studies such as the Orinda study, CLEERE study and SCORM study revealed a decrease in the RLP with older age in school children^9,11^. At baseline, RLP was highest in hyperopic eyes, followed by emmetropic eyes, and it was lowest myopic eye. In all refractive groups, the RLP steeply decreased up to the age of 10 years, while beyond that age, the rate of the reduction in RLP with older age decreased.

The present study showed that the RLP decreased from the age of 6 years to the age of 13 years, and then increased slightly to a plateau-like phase at the age range between 13 and 16 years. The age at which the RLP reached a stable state was in our study population as compared the populations of previous investigations at a later age (of 13 years)^11,30,31^. Potential reasons for the discrepancy between the study populations may be differences of in the refractive index of the lens and differences in the study populations, to mention only a few.

Interestingly, the lens thickness and the RLP were not significantly associated with the ethnic background. The findings obtained in our study agree with observations that children of European descent and Asian children did not differ significantly in the association of RLP with age^3,5,9^.

The RLP, corneal curvature radius and axial length are major optical components, which appear to play important roles in refractive development. During the process of emmetropization, axial elongation is coordinated with flattening of the cornea and decreases in RLP, so refractive errors will be relatively stable. However, the corneal power stabilizes after the age of 2 years^11^, so the change of RLP and axial length contributed to the development of refractive error. In our study, RLP was negatively associated with axial length (r =  − 0.700, *P* < 0.001) and corneal curvature radius (r =  − 0.140, *P* = 0.001). Excluding the effect of corneal refraction, RLP was still found to be negatively associated with axial length (r =  − 0.693, *P* < 0.001). These results were consistent with the previous longitudinal studies^9–11^. Between 1989 and 2007 in the Collaborative Longitudinal Evaluation of Ethnicity and Refractive Error Study, Mutti DO et al.^32^ found that the crystalline lens thinned, flattened, and lost power at similar rates for emmetropes and children who became myopic.With a follow-up of 3 to 6 years, Jos Rozema et al.^10^ found that the deceleration in RLP loss before myopia onsetuntil 1 year before myopia , rather than occurs simultaneously with onset as reported previously. Our finding with the logarithmic curve showed a similar tendency about association of RLP and the axial length.

The pattern of lens thinning in children is also related to myopia onset and progression^9^. More evident associations of RLP with axial length were observed in the non-myopes than in the myopes irrespective of age and sex. Girls have significantly thicker lens and greater RLP than boys. A Higher RLP was additionally associated with the shorter axial length and a thicker lens thickness. Recent cross-sectional and population-based studies with schoolchildren found that the RLP decreased markedly in children and adolescents accompanied by a thinning of lens up to the age 10 years, and after that, the decrease in RLP slowed down^17,18^. Our study revealed a significant difference between the refraction groups in lens thickness and RLP. The medium to marked hyperopia group had the lens, followed by the mild hyperopic group and the emmetropic group, and the lens was thinnest in the myopic group. The relationship between a lower RLP and longer axial length as found in our study in children has also been reported in population-based studies^17^.

Interestingly, the tendencies of longer axial length with older age are like the decrease in RLP in our study. The axial length increased more markedly in younger ages up to the age of approximately 14 years, and slowed down afterwards. It may imply that the change in axial length and in RLP were concomitant. the axial length develops quickly in younger ages, and the rate of development slows after the age of 12 years^33,34^.

Potential limitations of our study should be mentioned. Firstly, the lens power cannot be measured directly, which was calculated based on refraction, lens thickness, and other ocular biometric parameters with Bennett’s formula without equivalent refractive index, the study showed that the values of mean lens power calculated by Bennett’s formula had good agreement with that by phakometry methods^22,23^. Secondly, our study had a cross-sectional recruitment of participants so that we could not examine causal relationships between lens power or lens thickness as the main parameter and other parameters.

## Conclusion

In Chinese children, RLP with a mean of 24.85 ± 1.98D decreases with older age, male sex, longer axial length and thinner lens thickness. Lens thinning and potentially changes in the refractive index of the lens are causes of the changes in RLP during school years. Changes in RLP and axial length elongation are important players in the process of emmetropization and myopization.
